# Entrepreneurial ecosystems: economic, technological, and societal impacts

**DOI:** 10.1007/s10961-018-9690-4

**Published:** 2018-09-15

**Authors:** David B. Audretsch, James A. Cunningham, Donald F. Kuratko, Erik E. Lehmann, Matthias Menter

**Affiliations:** 1 School of Public and Environmental Affairs, Indiana University, Bloomington, IN 47405 USA; 2 Newcastle Business School, Northumbria University, Newcastle upon Tyne, UK; 3 Kelley School of Business, Indiana University, Bloomington, IN 47405 USA; 4 CCSE (Augsburg/Bergamo, It), University of Augsburg, Universitaetsstr. 16, 86159 Augsburg, Germany; 5 School of Economics and Business Administration, Friedrich Schiller University Jena, Carl-Zeiss-Str. 3, 07743 Jena, Germany

**Keywords:** Entrepreneurial ecosystem, Innovation ecosystem, National ecosystem, Ecosystem, Entrepreneurial firms, Regional clusters, University-industry collaborations, I23, L22, L26, R11

## Abstract

Despite the overwhelming use of the metaphor ‘ecosystem’ in academia, industry, policy, and management, exact definitions of what ‘ecosystems’ really comprise are scarce and often inconsistent. Existing vague descriptions in the literature do not consider the boundaries of respective agglomerations, hence, they impede the evaluation of performance and outcome measures of respective ecosystems. This special issue is a first attempt to trace the ‘ecosystem’ discussion back to its roots—the ancient *oikos*, coined by the Greek philosopher Hesiod (700 BC), and aims to critically reflect on the usage of the term ‘ecosystem’, briefly summarize the extant literature and grasp the main features of entrepreneurial ecosystems, namely the economic, technological, and societal dimensions of entrepreneurial ecosystems. We intend to focus on the key elements that characterize an ecosystem, and hence, untangle under what conditions entrepreneurial firms shape and influence economic, technological, and societal thinking within their ecosystem.

## Introduction

Entrepreneurs and new venture startups are increasing at an exponential rate across the globe. In light of this dramatic increase, there has been a surge in attempts to find greater ways to understand how to best assist these emergent ventures (Kuratko 2017). Thus, the rise of “entrepreneurial ecosystems” as organized attempts to establish environments that are conducive to increasing the success for newly established ventures. Yet, as this term has gained popularity, there remains a persistent question of exactly what is and what comprises an entrepreneurial ecosystem.

The metaphor ‘ecosystem’ has enjoyed increased popularity in academia, industry, policy, and management as a vehicle to describe, explain, advertise, and convey thoughts, frameworks, and opinions on how economic agents interact with their environment (Acs et al. 2017b; Colombo et al. 2017). The underlying idea is that firms do not just compete with each other through well-developed stand-alone strategies to achieve advantages over their rivals, uniquely relying on their own resources, knowledge, and capabilities. In a turbulent and hyperactive business world (D’Aveni 1994; D’Aveni et al. 2010; Minà et al. 2016), strategic and competitive advantages are increasingly based on shared resources, network externalities, knowledge spillovers, local endowments, and governmental support, creating a need for concepts beyond the firm specific competitive advantage approach (Porter 1990). Concepts, which consider not only those actors involved directly in the own firm specific value chain, like close suppliers, financiers or clients, but rather all factors which shape a firm’s value chain also in an indirect way, are therefore necessary. Such a view has to enrich the close competitive environment, rethinking existing causal relationships but also encompass physical and intangible assets, like infrastructures, institutions, sources of knowledge and human capital spillovers, and network effects (Audretsch et al. 2016; Jackson et al. 2017; Lehmann and Menter 2016, 2018; Kuratko et al. 2017). Drawing from biology, Moore (1993) pioneered the concept of business ecosystems as a cluster of interrelating actors, like different kinds of firms, universities, scientific parks, and public government that co-exist in a common setting and evolve together like creatures in their ecosystem do. A decade later, scholars have shown their interest in Moore’s work (1993, 1997) and the ‘ecosystem’ metaphor (Iansiti and Levien 2004; Adner et al. 2013; Isenberg 2010, 2014) has set off an avalanche in the ‘ecosystem’ research.

The overwhelming part of this literature is, explicitly or implicitly, aligned to the analogy of natural ecosystems “as a community of living organisms in conjunction with the nonliving components of their environment, where the ‘eco’ part of the word is assumed to be related to the environment and ‘system’ implies the function as a collection of related parts that function as a unit” (Smith and Smith 2015:19). Natural ecosystems can be of any size but usually encompass specific, limited geographic space. In an economic sense, an ecosystem consists of exogenously given components, the environment, and agents acting endogenously together as a system, linked by generating benefits from the interrelationship (Acs et al. 2016, 2017a). Like organisms in nature, different kinds of companies, multinational enterprises, small and medium sized and family enterprises, as well as entrepreneurial firms co-exist and co-evolve within their own ecosystem (Moore 1997).

Despite the rapidly growing literature on ‘ecosystems’, critical voices have challenged whether old wine is sold in new skins (Oh et al. 2016; Deog-Seong et al. 2016). This literature criticizes the inconsistent use of the term ‘ecosystem’ and its vague definition that adds no additional value to the scholarly discourse to existing concepts like ‘cluster initiatives’, ‘triple-helix initiatives’ or ‘public-private partnerships’ (Brown and Mason 2017). The metaphor ‘ecosystem’ reflects the tendency in academia to describe the old phenomenon of agglomeration effects of regions (urban, regional, national ecosystems) and industries (agricultural, chemical, manufacturing, media, finance ecosystems), i.e. clusters either of firms (business, entrepreneurial ecosystems) or activities (service, innovation, digital ecosystems) (see Bruns et al. 2017). The economic causes and consequences of agglomeration effects in academia date back at least to Alfred Marshall’s (1890) *Principles of Economics*, which were based on earlier work of Von Thünen (1826) and List (1841). More recent analyses include Nelson and Winter (1982). Since then, the concentration of activities within regional boundaries has fascinated scholars like Porter (1990) among others, and has stimulated considerable subsequent academic research (Acs et al. 2016, 2017a, b).

**Fig. 1:**
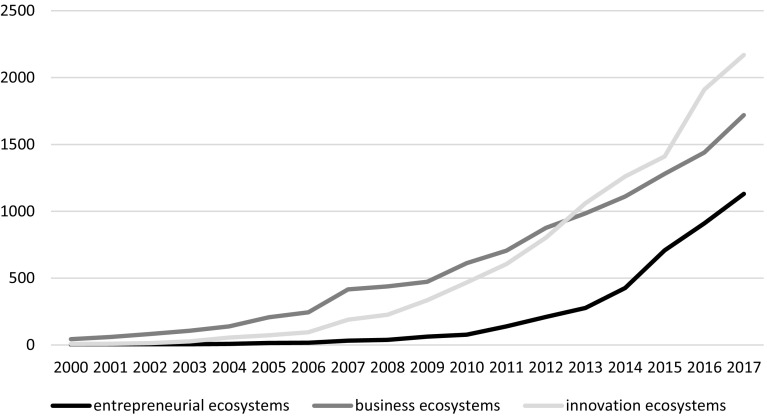
Studies focusing on ‘Ecosystems’ Source: Google Scholar

The attention on ecosystems and the intellectual ferment that it has generated in the last decades motivates the need to go back to lineages of the ecosystem metaphor and to shed some light to this exploding topic in academia (see Fig. 1). This special issue aims to briefly summarize the extant literature and to identify and articulate the main features of ecosystems, namely the economic, technological, and societal dimensions of ecosystems, as identified by Hesiod, who coined the term ‘ecosystem’ about 700 BC. We intend to focus on the key elements that characterize an ecosystem, and hence, untangle under what conditions entrepreneurial firms shape and influence economic, technological, and societal thinking within their ecosystem.

The remainder of this paper is structured as follows. The next section provides a brief overview of the increasing popularity of the ‘ecosystem’ metaphor. In section three, we focus on the Greek philosopher *Hesiod*, who coined the metaphor ‘ecosystem’ and we also refer to his analytical work on resource allocation and efficiency in a bounded area, the *oikos*. We take his framework as an organizing structure for the selected papers in this special issue, which are introduced in section four. Section five outlines fruitful avenues of future research on the topic ‘ecosystem’. A final section concludes.

## The popularity of ecosystems

Iansiti and Levien (2004) used the ‘ecosystems’ metaphor introduced by Moore (1993), and since then the ecosystem literature has gained increasing popularity in business, management, and policy. The number of publications drawing on the ‘ecosystem’ metaphor exploded since then (see Fig. 1), and national and local governments praise their countries or cities as constituting vibrant ‘ecosystems’, while managers of incubators, accelerators, or research parks are now managers of an ‘ecosystem’.

However, this explosion of articles comes at a cost. The problem any researcher faces in analyzing an ecosystem is one of definition. A question that is often asked but rarely answered, at least satisfactorily, is what actually constitutes a *bona fide* ecosystem. Is an ecosystem described by its economic activities, ‘innovation ecosystem’ (Acs et al. 2016), by its actors, ‘entrepreneurial ecosystems’ (Stam 2017a, b), or by its boundaries, ‘national ecosystems’ (Acs et al. 2017a, b)? The fact that this question is so difficult to answer may be one reason that there exists no ‘theory’ of the ecosystem and despite eluding a precise definition, the ecosystem literature suffers from a lack of development and chronic inconsistency. Any metaphor, like the ‘ecosystem’ metaphor can be pushed too far, and should be expected to elicit some critical responses. The ‘ecosystem’ is often treated as a metaphor for a special kind of network and network externalities, for a certain market or niche, for the complementarity of physical, human, and intellectual assets, or even for the externalities of certain activities. While applying the ‘ecosystem’ metaphor as an analogy to natural systems, critical questions still remain unanswered, like the spatial and virtual boundaries of an ‘ecosystem’, the governance aspects of ‘ecosystems’ (Rampersad 2016), or if ‘ecosystems’ are artificial or built from scratch (Colombo et al. 2017).

One explanation of the puzzle posed by the recent ascendance of ecosystems as a widely accepted and used metaphor in business and management is its general broadness and multi-disciplinarity (Autio et al. 2018). Only a small percentage of this research, if at all, is found in subfields where specific and codified knowledge, like mathematics, statistics, or economics, is required. The market for scientific concepts and their real life applications is characterized by trends, path dependencies and thus follows the concept of a life cycle. New concepts and metaphors in academia often gain a high attention in the initial state, attracting more and more interest, become an academic ‘hot spot’ and then almost burn up. Another explanation is the emergence of natural ecosystem in the recent public debate on sustainability and environmental protection, as something important for the planet. Thinking in terms of ‘ecosystems’ may also reflect the importance of a topic, like ‘business ecosystems’ (Moore 1993, 1997), ‘digital ecosystems’ (Sussan and Acs 2017), ‘university ecosystems’ (Hayter 2017; Wright et al. 2017; Meoli et al. 2017; Colombelli et al. 2017) or ‘financial ecosystems’ (Cumming et al. 2017; Ghio et al. 2017) to name just a few of them.

Metaphors are often an ‘invitation to see the world’ (Barrett and Coperrider 1990), serving as a means to ‘generate alternative social realities’ (Tsoukas 1991) and can be broadly defined as ‘a way of thinking and a way of seeing’ (Morgan 1983). Relying on metaphors is evident in the unrelenting search for new ideas and concepts from the most varied fields in business and management, in particular, when there is no substantial new to add (Kamoche et al. 2003). In this way, the metaphor of ‘entrepreneurial ecosystems’ has been mapped on to the environmental context in order to help us see entrepreneurial activities in a new light, thus generating useful insights into the phenomenon of ‘entrepreneurship’, and the terminus ‘entrepreneurship’ could be substituted for other ecosystems, like digital, business, or innovation.

While revealing certain truths, metaphors conceal other truths. The ‘entrepreneurial ecosystem’ metaphor is a vehicle for carrying us to larger insights into individual and collective action in the field of entrepreneurship, when the outcome of the action is rather unforeseen and unpredictable and the activity itself matters, at least for the interconnected actors. The instruments that entrepreneurs, policymakers, and managers of connected companies play are their metaphors for organizing the entrepreneurial ecosystem.

Entrepreneurial researchers have continued to demonstrate a healthy concern for creativity as they approach problems and issues in entrepreneurship. This is evident in the unrelenting search for new ideas and metaphors emanating from a broad and diverse range of research fields, such as the natural sciences. However, despite the many attempts to point out the analogy to the natural sciences, it is almost overseen that the metaphor of ‘ecosystems’ was coined by the Greek philosopher Hesiod (about 700 BC). Today, the thoughts of Hesiod are almost forgotten and the metaphor ‘ecosystem’ is used as an analogy of ‘natural ecosystems’, neglecting the historical roots of this terminus (Colombo et al. 2017). Hesiod described how resources should be allocated efficiently within households. In ancient Greece, such a household was named *oikos* (οἶκος) and the prefix ‘eco’ in ‘ecology’ and ‘economy’ originates from them. An *oikos* represented the basic economic unit of society in ancient Greek city-states, a self-sufficient and autarkic unit encompassing not only different people, materials, and goods, but also different activities under the governance of the despot as an institution (see Colombo et al. 2017). A well-developed *oikos* allocates resources in the way to support value creation for economic, technological, and societal benefits. This special issue is a first attempt to trace the ‘ecosystem’ discussion back to its roots—the ancient *oikos*. The critical dimensions, as identified by Hesiod nearly 3000 years ago, represent the organizing structure for the papers included in this special issue: the economic, technological, and societal dimensions of ecosystems.

## Economic, technological, and societal impacts of entrepreneurial ecosystems

An ecosystem is self-defined by boundaries, where the species within live together in autarky. The boundaries could be physically or not, but are associated with entry and exit barriers. The species, agents, absorb the necessary resources from the ecosystem and also produce critical resources for others, which spillover within and beyond the boundaries of the ecosystem.

*Economic impacts* refer to the anticipated increase in locational capital wealth and prosperity and how entrepreneurial ecosystems generate and create value. Due to the regional agglomeration of local factors and resources and their entrepreneurial exploitation as well as associated spillover effects, entrepreneurial ecosystems can contribute to a region’s vibrancy, sustainability, and viability. Ultimately, entrepreneurial ecosystems create competitive advantages and value for individual firms and sectors, and hence shape regional innovation outcomes (Cunningham et al. 2018). Economic impacts and successes also support the development of national and regional reputation of entrepreneurial ecosystems that, in turn contribute to attracting financial and human capital and other resources into the ecosystem. Ecosystems differ from each other by their production structure, as entrepreneurial ecosystems differ from business ecosystems, while the underlying production structure of entrepreneurial ecosystems remains a black box.

Ecosystems are often seen as an essential part of the economy, and the metaphor is often used to replace the traditional term of “markets”. Entrepreneurial ecosystems create and operate markets and thus a theory of the economic impact of entrepreneurial ecosystems should help to explain how entrepreneurial ecosystems arise and how they work. A general theory of the economics of entrepreneurial ecosystems differs from traditional neoclassical economics in a number of critical areas. Ecosystems are multi-firm, multi-product markets, with markets, which may almost exist in future times. Such multi-firm, multi-product combinations could not be captured by standard market economy theory, described by market structure, entry, and exit barriers, an underlying production structure (the technological pillar), and the degree of rivalry.

The degree of rivalry and competition is the second point where ecosystems differ from traditional markets. Ecosystems are by definition characterized by cooperation and network externalities to exceed quasi-rents and less by rivalry and competition and individual or firm level profit maximization. Another aspect refers to the kind of competition. Agents operating in ecosystems may be competitors for scarce resources and clients. Simple theories based on either price or quantity could not adequately describe and reflect the kind of competition. Standard economic theory also fails when profit maximizing could not be assumed for the agents involved, like public institutions. Then the question of the most relevant actors arises. This, finally, raises the question about governance issues (Colombo et al. 2017; Cunningham et al. 2017b). (Perfect) market theory assumes the ‘invisible hand’ governing the different agents by coordinating and motivating via the price mechanism.

*Technological impacts* relate to regional innovation mechanisms, i.e. the efficiency how innovation is pursued and realized. The efficient transformation of ideas and inventions to innovative products and services is crucial and dictates technology transfer and innovation processes. Some of these ideas have the potential to be disruptive in nature and in execution. Respective processes in turn influence the competitive positioning of a region and may encourage or discourage entrepreneurial behavior (Kuratko et al. 2017). Social impacts are associated with networks among a range of actors that participate in entrepreneurial ecosystems. Due to the co-existence of new ventures, small and medium sized as well as large firms (Bhawe and Zahra 2017), as well as universities and research institutions (Audretsch and Link 2017) within such ecosystems, respective agglomerations or networks (Audretsch and Belitski 2017) have to be governed and organized to enable efficient knowledge flows and value creation processes within these ecosystems (Cunningham et al. 2018; Colombo et al. 2017). While the almost descriptive literature of entrepreneurial ecosystems puts a lot of emphasis on the role of intermediate inputs—the local labor force, production and absorption of spillovers, and non-market interactions—the technological dimension of entrepreneurial ecosystems needs a more precise formulation and modulation of how inputs are transformed to outputs, which parts of the pillars are complementary choice variables and which are substitutes (Roberts 2004).

It may be tempting to specify an aggregate production function, like a standard growth model that directly links input to output factors as customary to measure total factor productivity. In the case of entrepreneurial ecosystems, such a standard simplification is not adequate for several reasons. The first is because the input-output relations are often multiple, and multiple inputs create multiple outputs. The second is because inputs and outputs cannot be clearly defined. Entrepreneurial firms and new venture creation serve as important inputs to entrepreneurial ecosystems in order to penetrate the knowledge filter but are also performance measures (Stam 2017b). Another aspect that is almost neglected relates to the boundaries of an (entrepreneurial) ecosystem: whether there exists a spatial dimension of the ecosystem to define, who is in and who is not, whether the dimension is real or virtual. Analyzing the economic impact does not make sense when the boundaries are not clearly defined (Stam 2015).

Finally, an entrepreneurial ecosystem, as any ecosystem, has to fulfill two tasks—to generate value for the ecosystem and to distribute the value among the members of the ecosystem (Clarysse et al. 2014; Stephen et al. 2012; Vargo and Lusch 2010). While the economic and technological dimensions try to provide answers on the creation of value in entrepreneurial ecosystems, the third pillar, the societal dimension is concerned about the second question.

*Societal impacts* therefore refer not only to monetary but also to non-monetary outcomes, i.e. the social boundaries among entrepreneurial ecosystem actors. The social benefits can spillover into the delivery of new products and services that are beneficial for society. The benefits and impacts may also relate to collective value creation and public good impacts (see Cunningham et al. 2018). The papers in this special issue fit within these three pillars of entrepreneurial ecosystems, as briefly introduced in the next section.

## Themes and contributions

The first paper, by Fudickar and Hottenrott (2018), is entitled *Public Research and the Innovation Performance of New Technology Based Firms*. Publicly funded scientific research in entrepreneurial ecosystems has gained an increased importance for science and entrepreneurship policy. As academic research is expected to flow and spillover across an entrepreneurial ecosystem, this study examines the economic impact of direct interactions with public research institutions on new technology-based firms (NTBFs) innovation success. Taking a large sample of NTBFs in Germany, the authors find that those firms engaging in such knowledge interactions are more likely to introduce new products and services to the market. Highlighting the importance of absorptive capacities, the authors further suggest that continuous informal interactions complement formal ones in the absence of own R&D activity. The results thus support the argument that public research plays a key role in knowledge and technology transfer and eventually for innovation in entrepreneurial ecosystems.

The second paper entitled *The Organisational and Geographic Diversity and Innovation Potential of EU*-*funded Research Networks* by Nepelski et al. (2018), also investigates how public funding of research affects the systemic conditions of entrepreneurial ecosystems. Utilizing a sample of 603 collaborative research projects supported by European Commission Framework Programmes (FP), the authors assess the innovation outcomes of FP projects in ICT and find that innovations developed by research networks with a higher organisational diversity have more commercial potential. Thus, policies improving systemic framework conditions of entrepreneurship ecosystems through the creation of institutionally diverse research networks can have beneficial effects on the commercialization potential of innovations developed in FP projects. In contrast, research networks with a wider range of internationally dispersed research partners seem to be more likely to have less innovation potential.

Universities as key actors within the entrepreneurial ecosystems not only disseminate knowledge, but also commercialize knowledge themselves through academic spinoffs. Civera et al. (2018) study the post-entry internationalization of academic spinoffs in terms of international sales in their paper *Do Academic Spinoffs Internationalize?*. Matching a sample of 508 Italian academic spinoffs established between 1999 and 2014 with 404 comparable non-academic innovative start-ups, the authors find that university spinoffs are more prone to internationalize than their non-academic counterparts. Considering the economic, societal, and technological impact of academic spinoffs, the authors argue that internationalized universities might act as intermediaries of internationalization, representing the feeders of entrepreneurial ecosystems that provide trained entrepreneurs with access to their knowledge and technologies.

The commercialization of knowledge as well as the associated transformation of inventions to innovations is a crucial measure of success within entrepreneurial ecosystems. In their study *Patent*-*based Investment Funds: From invention to innovation*, Jarchow and Röhm (2018) investigate the phenomenon of patent-based investment funds as a new type of intermediary in the knowledge spillover process, which could facilitate the transformation from invention to innovation within entrepreneurial ecosystems. Using a qualitative research design, the authors find common characteristics of funds’ activities, which decrease knowledge filters and fill the financing gap in the early stages of technology development, and propose a classification of commercialization strategies, linking those to a specific set of invention characteristics. This paper thus disentangles part of the technological impact of patent-based investment funds and emphasizes the need to potentially adjust path-specific activities according to the technology’s characteristics in order to choose the best way of commercialization within an entrepreneurial ecosystem.

In their study *Stimulating Academic Patenting in a University Ecosystem: An Agent*-*Based Simulation Approach*, Backs et al. (2018) explicitly focus on the actual patenting process within a university ecosystem. As institutions within university ecosystems such as technology transfer offices shall facilitate and promote patenting activities and entrepreneurship, respective impacts on the commercialization process are crucial to identify. Utilizing an agent-based simulation approach, the authors propose an evaluation measure that is meant to stimulate academic patenting and, subsequently, the foundation of spin-off companies relying on such patents. These technological impacts of agent-based simulations in turn affect perceived opportunities taken by researchers and practitioners and that ultimately foster university-industry cooperation and academic spin-offs.

The next paper *Having Friends in High Places: An Empirical Study on Social Boundaries in Entrepreneurial Ecosystems* by Neumeyer and Santos (2018), studies the societal impacts of entrepreneurial ecosystems. Advancing a social network perspective, the authors model the entrepreneurial ecosystems of two municipalities through a diverse network of entrepreneurs, investors, and institutional leaders. The results suggest that entrepreneurial ecosystems consist of different social clusters, forming boundaries along venture type (e.g. high-growth, lifestyle), type of support institution (e.g. university, government agency), gender, and ethnicity. This study thus supports the increasingly prominent role that the underlying structure assumes in dynamic ecosystem behavior, but raises new questions about its temporal and contextual boundary conditions.

Our final paper *Evaluating and Comparing Entrepreneurial Ecosystems using SMAA and SMAA*-*S* by Corrente et al. (2018), focuses on the entrepreneurial ecosystem as a set of interdependent and coordinated factors in a territory enabling entrepreneurship. The paper addresses the lack of empirical analysis that discriminates between factors according to their importance. Producing a probabilistic ranking through the application of an accurate, robust, and reliable measurement technique, namely Stochastic Multicriteria Acceptability Analysis (SMAA), to obtain a comparison among entrepreneurial ecosystems, the results show that the most relevant entrepreneurial ecosystem factors enabling the birth and activity of high-growth start-ups, and so impacting on technology, economy and society, can be identified in cultural and social norms, government programs, and internal market dynamics.

## Future avenues of research

The papers in this special issue have provided further empirical insights into the economic, technological and societal impacts of entrepreneurial ecosystems. Future research should specifically focus on the creation, governance, and sustainability of entrepreneurial ecosystems. How are entrepreneurial ecosystems actually created and how is self-sufficiency achieved as part of an *oikos* that support the distributed value creation for economic, technological, and societal benefits? Moreover, how replicable are entrepreneurial ecosystems across different sectoral, technology, geographic, regulatory and legal environments.

Another challenging and important issue within the ecosystem literature refers to the actual boundaries of entrepreneurial ecosystems. A better and deeper understanding of the boundaries is essential to appropriately evaluating the performance, output, and impact of respective ecosystems. As existing studies have mainly considered one specific context, future studies should apply cross-country, cross-industry, cross-societal research designs. In addition to that, advancing the array of appropriate evaluation tools and methodologies to evaluate the performance, vibrancy, stability and resilience of an entrepreneurial ecosystem is necessary.

There is a need for micro level analysis of entrepreneurial ecosystems and as part of an *oikos* how different actors, materials, and goods can be organized in a sustainable and optimal manner—formally and informally—to achieve beneficial outcomes individually and collectively (see Cunningham and O’Reilly 2018). For example, is it the same group of individual actors that drives the development of ecosystems, irrespective of the geographical context, or do external shocks or perceived or actual threats instigate the creation of an entrepreneurial ecosystem? What role do individuals play in shaping the creation, evolution and sustainability of entrepreneurial ecosystems? For example how do scientists in the principal investigator role for large publicly funded research drive cohesion and collective value creation for the benefit of all ecosystem actors? How do TTO directors, public research lab directors and those in leadership position in state and regional support agencies influence the policy, specific policy instruments to support embryonic ecosystems? What roles do trade and industry associations play at a micro level in shaping perceptions of entrepreneurial ecosystem?

An interesting and challenging research avenue would be to explore the entry and failure of firms in established entrepreneurial ecosystems. The entry of new firms can be beneficial to building the capability and capacity of entrepreneurial ecosystems but may have some unintended consequences. Similarly, firms that fail in an entrepreneurial ecosystem can undermine the perceived reputation success and potentially destroy individual and collective value. A potential benefit of firm failure is that it frees up resources and capital to be deployed on new opportunities that will create value. However, from the growing body of research on entrepreneurial failure (see Cardon et al 2011; Simmons et al. 2018; Walsh and Cunningham 2016) it has individual impacts for entrepreneurs. Future research could examine some of these issues and how regenerative entrepreneurs utilize the entrepreneurial ecosystem to create a new business venture? Moreover, how do serial entrepreneurs also leverage entrepreneurial ecosystems to exploit market opportunities? Empirical studies should further investigate how and when entrepreneurial ecosystems.

The societal dimensions and impact of entrepreneurial systems are under researched. There is a rich array of themes that need to be explored as the societal impacts tend to be considered as less important than economic or technological impacts. Some potential areas of research could focus on how social enterprises support and contribute to the governance and functioning of entrepreneurial ecosystems. How does entrepreneurial diversity—immigrant, youth, people with disability, female etc (see Bewaji et al. 2015; Carter et al 2015; Van Niekerk et al. 2006)—contribute to the creation and sustainability of entrepreneurial ecosystems? How does an entrepreneurial ecosystem benefit them in realizing their entrepreneurial ambitions?

More critical analyses are needed in order to streamline the proliferated discussion on entrepreneurial ecosystems and add further to the literature beyond selling old wine in new skins. This requires new theoretical developments drawn from a wider range of disciplines that provide further underpinning with respect to the creation, evolution, and impact of entrepreneurial ecosystems. This is turn requires further empirical studies to test these theoretical perspectives and to do so using a wider range of methodological approaches (see Cunningham et al. 2017a).

## Conclusion

Despite the rapidly growing literature on ‘ecosystems’, there is literature that criticizes the inconsistent use of the term ‘ecosystem’ and its vague definition that adds no additional value to the scholarly discourse. Therefore, we focused on the key elements that characterize an ecosystem, and hence, attempted to untangle under what conditions entrepreneurial firms shape and influence economic, technological, and societal thinking within their ecosystem. This special issue serves as an attempt to address existing inconsistencies in the usage of the term ecosystem, stimulate a more critical reflection on entrepreneurial ecosystems as well as highlight the economic, technological as well as societal impacts of entrepreneurial ecosystems. Due to the explosion of studies related to ecosystems, an assessment of recent developments and an understanding of the state-of-the-art is crucial in order to further add to the existing literature on entrepreneurial ecosystems. It is our hope that this special issue shall thus serve as a guidance and critical reflection for scholars interested in this field of study.
